# A new method for the treatment of myocardial ischemia-reperfusion injury based on γδT cell-mediated immune response

**DOI:** 10.3389/fcvm.2023.1219316

**Published:** 2023-08-03

**Authors:** Wei Luo, Xiaohong Bian, Xiaona Liu, Wenchao Zhang, Qing Xie, Limin Feng

**Affiliations:** ^1^ Graduate School, Tianjin University of Traditional Chinese Medicine, Tianjin, China; ^2^ Department of Cardiology, The Second Affiliated Hospital of Tianjin University of Traditional Chinese Medicine, Tianjin, China

**Keywords:** myocardial ischemia reperfusion injury, immune response, T lymphocytes, γδT cell, cytokine, IL-17

## Abstract

Acute myocardial ischemia is a disease with high morbidity and mortality, and re-perfusion is currently the best intervention. However, re-perfusion may lead to further myocardial injury and increase the area of myocardial infarction. The mechanism of myocardial ischemia-re-perfusion injury is complex, but with more in-depth study, it has been proved that the immune system plays an important role in the process of MIRI. Among them, the γδT cell population has received increasing attention as the main early source of IL-17A in many immune response models. Because γδT cells have the characteristics of linking innate immunity and adaptive immunity,they can rapidly produce IL-17A and produce subsequent immune killing of cardiomyocytes. It can be seen that γδT cells play an important role in MIRI. Therefore, here we review the research progress of immune response in myocardial ischemia-re-perfusion injury, the key characteristics of γδT cells and the role of rapidly produced IL-17 in myocardial ischemia-re-perfusion injury, and propose relevant treatment strategies and prospects for myocardial repair, in order to provide new ideas and methods for clinical treatment of myocardial ischemia-re-perfusion injury.

## 1. Introduction

Acute myocardial infarction (AMI) is a cardiovascular disease that seriously threatens human health worldwide. Re-perfusion is the preferred treatment strategy for acute myocardial ischemia (1–4). Thrombolysis or percutaneous coronary intervention (PCI) is currently the most effective treatment to reduce ischemic injury and limit infarct size, thereby preventing ventricular remodeling, improving cardiac function, reducing arrhythmia, and effectively reducing mortality (5, 6). However, after the re-perfusion of ischemic myocardium, the abnormal changes of myocardial morphology and function caused by re-perfusion may lead to the necrosis of some ischemic myocardial cells, even more serious than the damage caused by ischemia alone, thus increasing the area of myocardial infarction. Although the best re-perfusion therapy is obtained after AMI, nearly 10% of patients still die, and the incidence of heart failure after AMI is as high as 25%, which seriously affects the effect of ischemic myocardial re-perfusion therapy (7). Therefore, exploring the pathogenesis of myocardial ischemia-re-perfusion injury and taking targeted prevention and control measures have become one of the urgent problems to be solved in cardiovascular clinical treatment, and have important clinical significance for effectively reducing mortality.

At present, it is believed that the mechanism of myocardial ischemia-re-perfusion injury is complex, which involves oxygen free radical injury (8, 9), calcium overload (10, 11), oxidative stress (12, 13), reactive oxygen species production (14), immune cells (15), endothelial cell dysfunction (16), autophagy, ferroptosis and other aspects. With the progress of immunological research, more and more evidence shows that immune response plays a central role in the pathological process of myocardial ischemia-re-perfusion injury. The enhancement of immune response mediated by γδT lymphocytes may aggravate the degree of myocardial ischemia-re-perfusion injury.Therefore, in this review, we discuss the role of immune response in myocardial ischemia-re-perfusion injury and the key characteristics and functions of γδT cells and their potential in the treatment of myocardial ischemia-re-perfusion injury.

## 2. Overview of the immune system

The immune system is a complex network composed of various immune cells, signaling pathways and effector molecules. It can be divided into two different types: innate immunity and adaptive immunity. Each type can recognize and respond to a variety of antigens. The immune system is activated under conditions such as tissue damage, infection or genotoxic stress, resulting in innate immune responses.

### 2.1. Innate immunity

The evolutionary innate immune system is much older than the adaptive immune system. It is composed of the complement system and different types of immune cells, including phagocytes (macrophages, neutrophils), antigen presenting cells (dendritic cells) and so on (17). The first line of defense for immune defense is based on the detection of pathogen-associated molecular patterns that cause toxic and inflammatory responses. Pattern recognition receptors in immune cells are activated when they respond to conserved motifs of invading pathogens and non-self elements (pathogen-associated molecular patterns). PPRs may also respond to endogenous molecular patterns released during cell injury or death, namely damage-associated molecular patterns (DAMPs), and subsequently induce aseptic inflammation. Among them, dendritic cells can further activate the adaptive immune response through antigen presentation. In MIRI, dendritic cells are considered to be the source of DAMPs release from cardiomyocytes after myocardial ischemia. The mechanism is that NADPH oxidase-dependent super-oxide production in dendritic cells is enhanced, resulting in the formation of highly active γ-ketoaldehyde. These compounds rapidly form their own protein adducts, which are treated by dendritic cells and presented as DAMPs, leading to vascular dysfunction.

### 2.2. Adaptive immunity

An adaptive immune system in which pathogenic exposure confers long-term defensive memory to host organisms, including T lymphocytes and B lymphocytes. B lymphocytes mainly detect and process antigens, and further differentiate in plasma cells to produce antibodies (immunoglobulins) to resist the invasion of harmful antigens and participate in humoral immunity (18). T lymphocytes mediate cellular immunity and assist B lymphocytes to produce antibodies (19). In a relatively new immune model, it has been shown that the immune system can respond to “danger signals”, both self and non-self. Exogenous “danger signals”, pathogen-associated molecular patterns (PAMPs), are highly conserved gene sequences in microbial pathogens, such as lipopolysaccharide (LPS), peptidoglycan, bacterial lipid oleic acid and flagellin. Endogenous “danger signals”, namely damage-associated molecular patterns (DAMPs), may come from poor or damaged cells, such as ischemic cardiomyocytes and infarcted cardiomyocytes. Both PAMPs and DAMPs can activate the immune system through PRRs and trigger innate and adaptive immunity. In addition, T lymphocytes also have the characteristics of inhibiting immune response and maintaining self-tolerance. The mechanisms of its inhibitory function include inhibition of cytokine secretion (IL-10, TGF-β and IL-35), direct cytolysis of effector T cells, destruction of metabolism through tryptophan decomposition products, IL-2 deprivation and direct interference with co-stimulation through cytotoxic T lymphocyte-associated protein 4 (CTLA-4) expression (20).

Although these two systems mainly protect organisms from invading pathogens, under disease conditions, their own cells may be the target of destruction, and invading immune cells can cause damage to the host they intend to protect. There are many different links linking the innate and adaptive immune responses, including the complement system, and involving cell types with two systemic functional characteristics, including B1 cells and γδT cells (21, 22).

## 3. The role of T lymphocyte sub-types and their mediated immune response in MIRI

Recent studies have shown that myocardial ischemia-re-perfusion injury is a complex process involving metabolic and immune factors. Immune response plays a central role in the pathological process of myocardial ischemia-re-perfusion injury. Immune response regulates the whole process of myocardial ischemia-re-perfusion injury by recruiting and activating related immune cells, innate immune system and adaptive immune system. T lymphocyte-mediated immune response plays an important role in myocardial ischemia-re-perfusion injury. Therefore, various T lymphocyte sub-types have been widely studied, including NKT cells, TH17 cells, γδT cells, CD4^ + ^T cells and CD8^ + ^T cells, among which γδT cells play an important role in myocardial ischemia-re-perfusion injury.

Adaptive immune response involved in myocardial ischemia/re-perfusion is a local inflammatory response based on cellular immunity. Studies have found that the early stage of ischemia-re-perfusion (IR) is dominated by acute inflammatory response. In myocardial ischemia-re-perfusion injury, myocardial tissue in the damaged area is mainly infiltrated with CD4^ + ^T cells as the main effector cells, which can infiltrate into the infarct area 2 min after re-perfusion and participate in sustained and stable myocardial injury. Primitive CD4^ + ^T lymphocytes can differentiate into helper T cells (Th cells) subsets and Foxp3 + regulatory T cells (Treg cells) under the action of various factors. Th cell subsets are mainly Th17 cells. Th17 cells and Treg cell subsets are the main participants in the immune inflammatory response. TH17 cells are a pro-inflammatory subset that promotes autoimmune and tissue damage. Th cells mainly secrete inflammatory cytokines such as interleukin (IL) −2, interferon (IFN) -γ, tumor necrosis factor (TNF) -β.Cytokines have pro-apoptotic effects in mouse myocardial I/R cardiomyocytes, and increase neutrophil infiltration by enhancing the production of chemokines (including CXCL1, CXCL2 and CXCL6), promote inflammatory response, and cause myocardial cell damage. On the contrary, Treg cells have immunosuppressive effects and can prevent autoimmunity. They not only regulate adaptive immunity by inhibiting the proliferation and function of effector T cells, but also regulate innate immunity by inhibiting macrophage inflammatory phenotype and neutrophil function.

At the same time, during myocardial ischemia-re-perfusion injury, endogenous ligands released by tissue damage activate Toll-like receptors, NOD-like receptors, C-type lectin receptors, and RIG-1-like receptors (23), thereby initiating natural immune responses (24), and then transcriptionally regulate the production of pro-inflammatory mediators, including cytokines, chemokines, and adhesion molecules, leading to tissue inflammation, thereby aggravating myocardial ischemia-re-perfusion injury.

**Figure 1:**
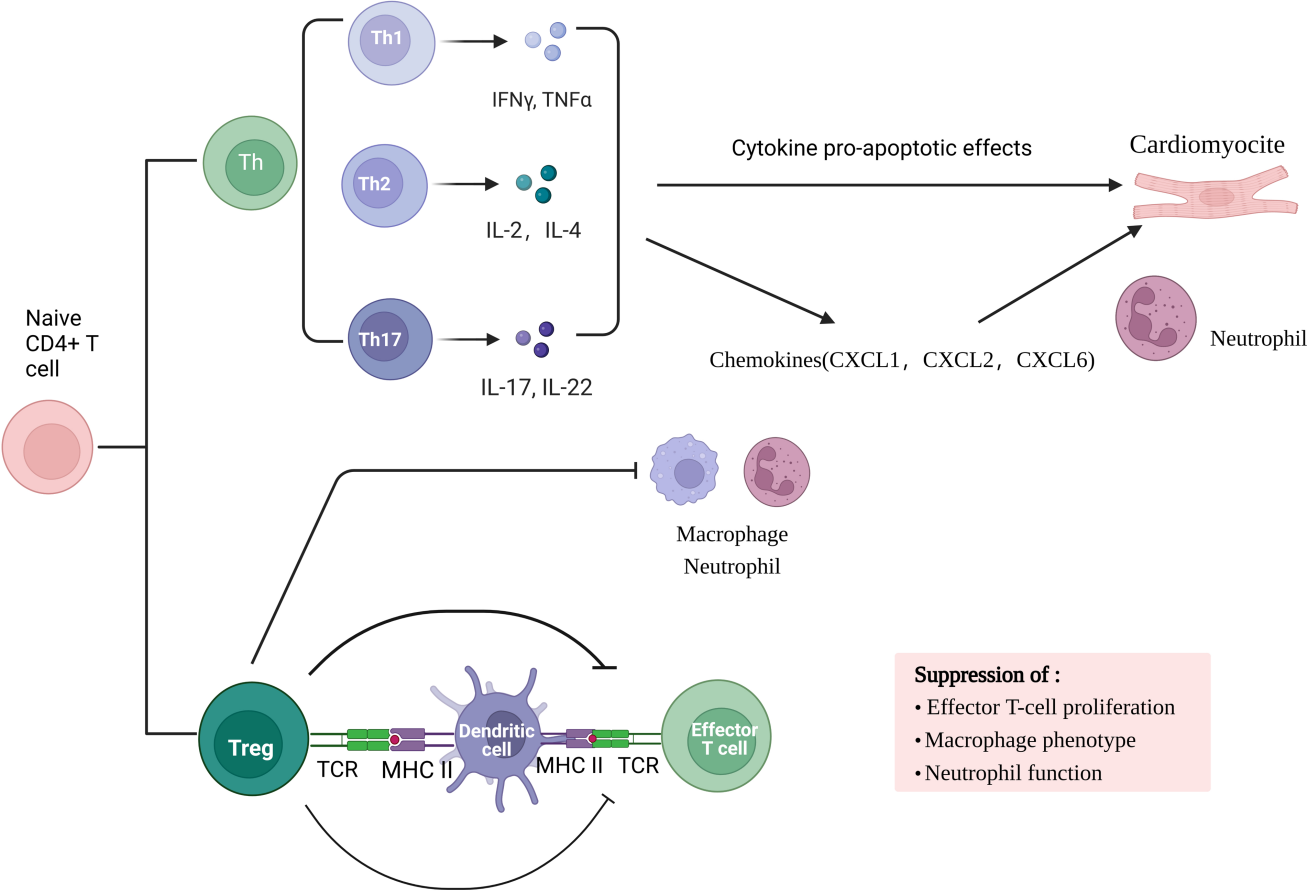
The role of T lymphocyte sub-types and their mediated immune response in MIRI.

In conclusion, in the process of myocardial ischemia-re-perfusion injury, metabolic abnormalities during hypoxia-re-oxygenation release dangerous signals, activate the body's natural immune system, activate TLR, complement system and mast cells, and subsequently recruit a large number of neutrophils and monocytes. Pro-inflammatory factors and oxygen free radicals are involved in the amplification of inflammatory reactions. Any part of the system overreaction will aggravate tissue damage. Most T cell subsets mainly contribute to the antigen-specific effects and memory stages of immunity, but γδT cells combine the characteristics of adaptive immunity with rapid innate immune responses, allowing them to be in the initial stage of immune response Figure 1.

## 4. Overview of γδT cells

### 4.1. Characteristics of γδT cells

According to the surface expression of T cell receptor (TCR), T cells are divided into two main groups: αβT cells and γδT cells. Different from αβ T cell population, the surface T cell receptors of γδT cells are composed of γ chain and δ chain. γδT cells are highly heterogeneous cells with various sub-types, variable phenotypes and different biological characteristics among sub-types. γδT cells are T cells that perform innate immune function. Compared with traditional αβT cells, γδT cells have unique properties that link innate immunity and adaptive immunity. They play an important role in the development of infection, tumor and autoimmune diseases. The antigen recognition of mouse or human γδT cells does not require the presentation of major histocompatibility complex (MHC) class I or II antigens (25). Activated γδT cells can enter the activated state within a few minutes after antigen stimulation (26). Activated γδT cells affect other immune cells by producing cytokines and cytotoxic multiple effector functions, regulating antigen presentation functions, thereby enhancing the immune response to dangerous signals formed by invading pathogens or “own” cells.

### 4.2. The classification and function of γδT cells

**Figure 2:**
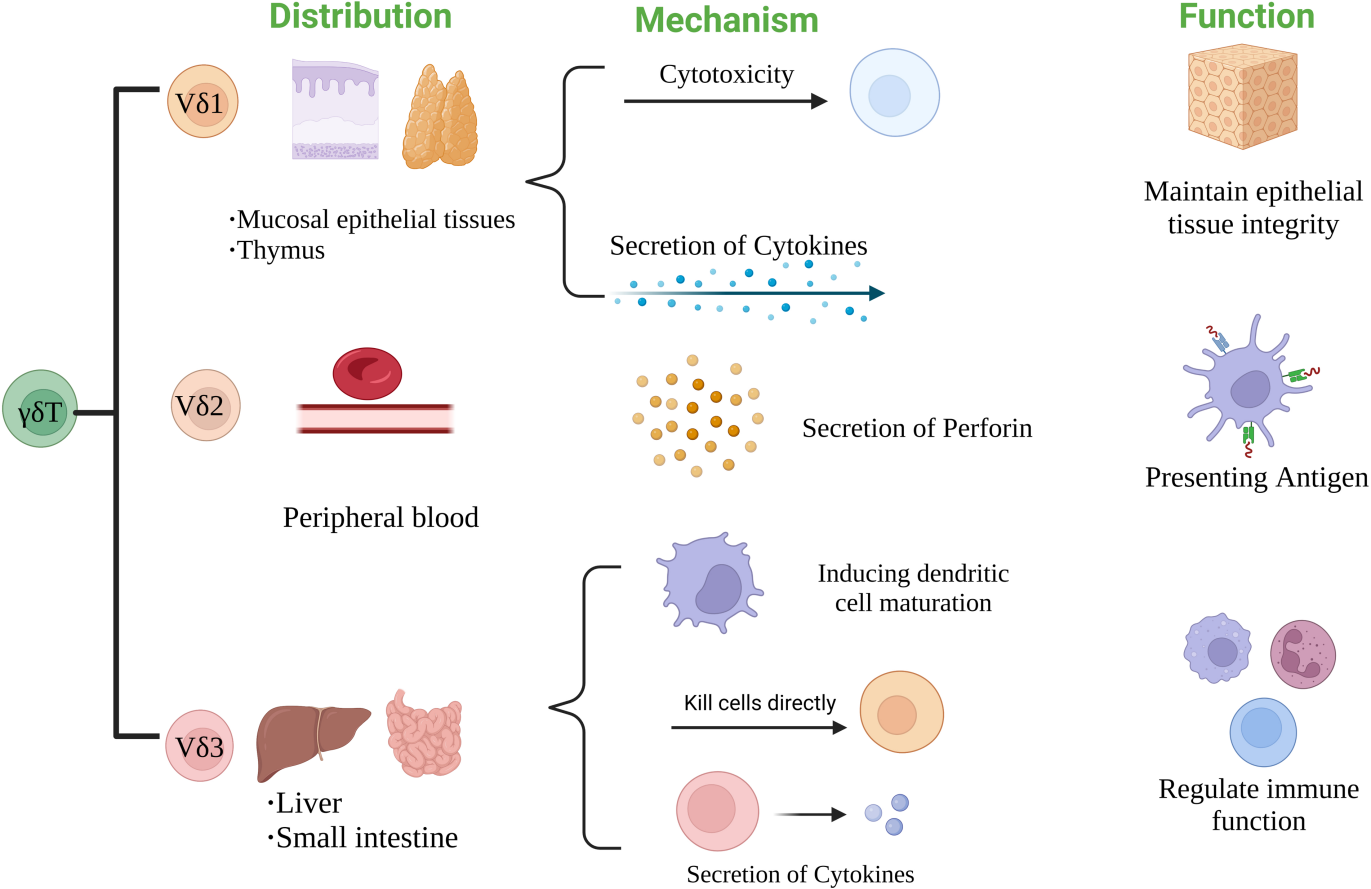
The classification of TCR δ chain structure of γδT cells.

According to the structure of TCR δ chain, γδT cells can be divided into three subgroups: Vδ1, Vδ2 and Vδ3 γδT cells. The distribution and function of each subgroup are also different (27). Vδ1 γδT cells are mainly distributed in mucosal tissues such as skin and small intestine. They can respond to stress antigens of epithelial cells (28) and participate in maintaining epithelial tissue integrity in the face of injury, infection or transformation (29). Vδ2 γδT cells are mainly present in peripheral blood, accounting for 50%–90% of peripheral γδT cells, and are the main γδT cells involved in blood circulation. Activated Vδ2T cells can be used as professional antigen presenting cells (APC) (30), such as antigen presentation, costimulatory and adhesion molecules expression, including MHC-II, CD80 and CD86 (31). Vδ3 γδT cells are mainly present in the liver and intestinal epithelium, accounting for about 0.2% of circulating T cells. They can kill CD1d target cells, release cytokines such as Th1, Th2 and Th17, and induce dendritic cells (DC) to mature into APC^+^ (32) Figure 2.

**Figure 3:**
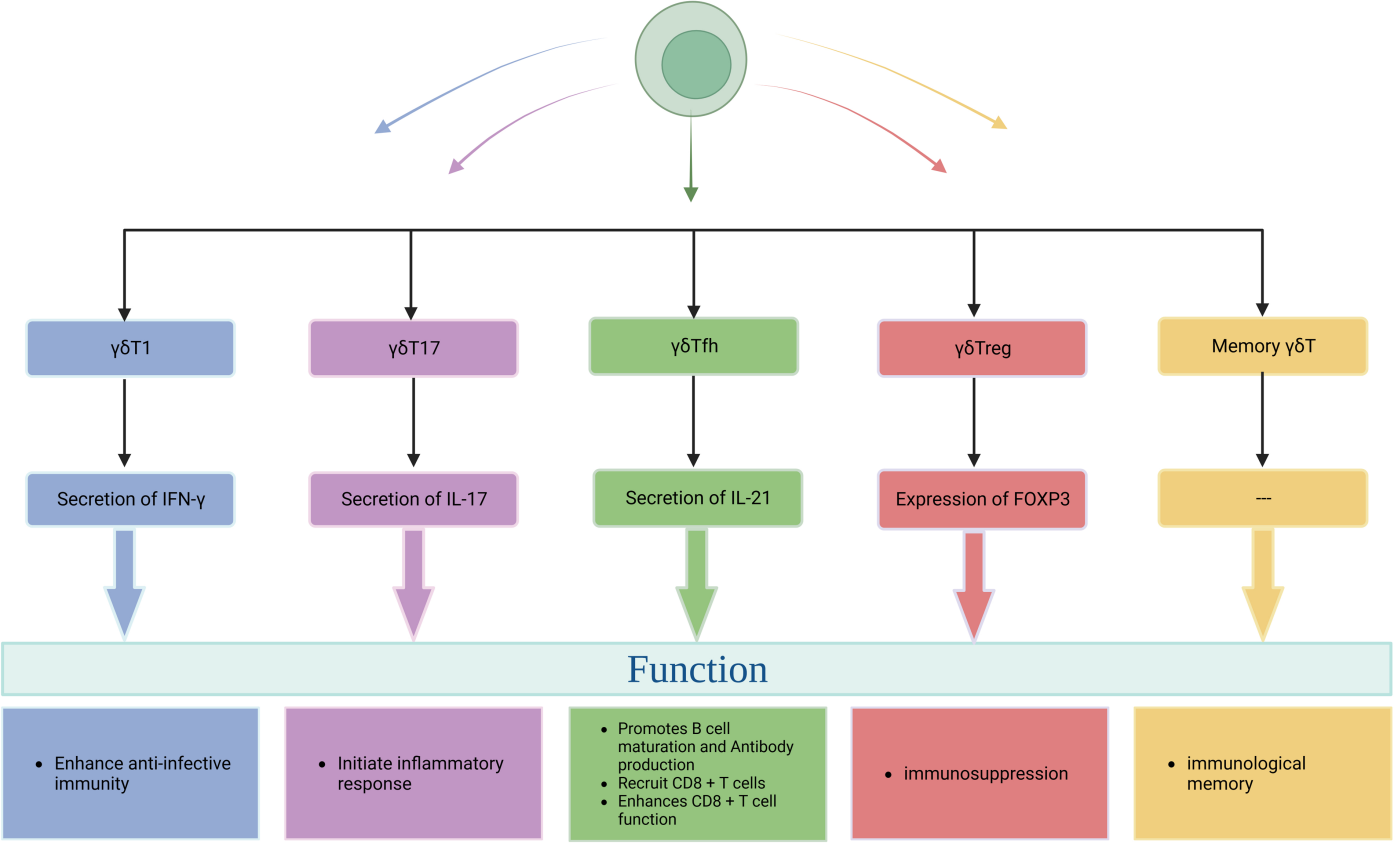
The functional classification of γδT cells.

They were classified according to the function of γδT cells. The heterogeneity of γδT cell subsets determines the diversity of their functions, so it can also be divided into γδT1 (IFN-γ^+^ γδT), γδT17, follicular helper γδT (γδTfh), regulatory γδT (γδTreg), memory γδT (memoryγδT) cells, hMSH2-specific γδT cells and recently discovered IL-6-secreting γδT cells. Among them, hMSH2-specific γδT cells and IL-6-secreting γδT cells have not been reported in the literature, and their structural and functional characteristics are still in the preliminary stage. γδT1 (IFN-γ^+^ γδT) cells can secrete IFN-γ, which can enhance cell-mediated anti-infective immunity. γδT17 cells can secrete IL-17, which plays an important role in initiating inflammatory response, regulating the expansion and recruitment of neutrophils and monocytes, and plays an important role in the initial stage of various inflammatory responses (33, 34). γδTfh cells can promote the maturation of B cells and the ability to produce antibodies; in addition, in some chronic infections and tumors, γδTfh cells can enhance the effector function of CD8^ + ^T cells and the proliferation of CD8^ + ^T cells by secreting IL-21 (35, 36); γδTreg cells can express a specific transcription factor FOXP3 and have immunosuppressive function (37); memory γδT cells have the characteristics of immune memory and can rapidly produce immune response after receiving the same antigen stimulation again (38). In summary, each γδT cell subset plays different roles in the human immune system and has potential clinical value Figure 3.

### 4.3. The immune regulation of γδT cells is bidirectional

#### 4.3.1. γδt cell-mediated immune response

γδT cells have the characteristics of congenital and adaptive immune cells, and can be activated only through APC cytokine signaling without homologous TCR ligands (39). When activated, γδT cells produce Th1 and/or Th2 inflammatory cytokines and IL-17 (40, 41), and then induce the inflammatory response of adaptive effector cells (42). These characteristics make γδT cells an effective effector T cell-mediated immune cascade in inflammatory tissues (43), and γδT cells can exert cytotoxic mediated killing of multiple target cells through Fas/Fas ligands (44). In addition, γδT cells can also play a variety of roles in the response to infection, including direct antibacterial effects, recruitment of innate immune cells (such as neutrophils, macrophages) and activation of adaptive immunity (45).

In the anti-tumor effect, studies have found that γδT cells are involved in several types of cancer, including breast cancer, stomach, colorectal cancer, hematological malignancies, and glioblastoma (46). As a key participant in anti-tumor immune response, γδT cells have the ability to produce a large number of pro-inflammatory cytokines and directly mediate cell lysis of various tumor types (47). At the same time, studies have confirmed that Vδ1 and Vδ2 subsets play an important role in the tumor immunity of γδT cells, mainly through natural killer cell receptors to identify tumor cells (48), and can play an anti-tumor role through direct contact and secretion of cytokines (49). Vδ1T cells kill cancer cells by up-regulating the expression of CD69, CD107a, perforin, granzyme B, TRAIL and CD57 (50). Vδ2T cells can directly kill or induce tumor cell apoptosis after TCR-dependent activation, and can also induce neutrophil infiltration to tumor sites or affect other immune cells to exert anti-tumor effects by secreting cytokines (51).

γδT cells play an important role in re-perfusion injury. In intestinal IRI, γδT cells are involved in the initiation and continuation of the initial inflammatory response as a mediator to promote the acute inflammatory response of intestinal IRI (52, 53); the lack of γδT cells can improve the production of pro-inflammatory cytokines, reduce neutrophil recruitment and distant organ damage (54, 55). In renal IRI, γδT cells mediate innate and adaptive immune responses during the first 72 h of renal IRI, and the absence of γδT cells will delay the inflammatory response in renal IRI (56–58). In brain IRI, studies have shown that IL-23 and IL-17 play a key role in the evolution of cerebral infarction and accompanying neurological dysfunction. IL-23 plays a role in the direct stage of cerebral IRI, while IL-17 plays an important role in the delayed stage of cerebral IRI. IL-23 secreted by activated macrophages can drive γδT cells to produce IL-17, which further increases neuroinflammation and secondary damage after intracerebral hemorrhage (59). Another study has also shown that the expression of IL-23 is mainly derived from infiltrating macrophages, and is an important inducer of IL-17 production by γδT cells in the delayed phase of cerebral ischemia in mice, and γδT cells that produce IL-17 play an important role in late cerebral infarction. Increased expression of IL-17 aggravates secondary brain injury after intracerebral hemorrhage, and γδT cells are the main source of IL-17 in the hemorrhagic hemisphere (60, 61). It has been found in liver IRI that IL-17A produced by liver γδT cells can lead to liver cell damage and enhanced liver inflammation in animals (62).

#### 4.3.2. Immunosuppressive effect of γδT cells

In systemic inflammatory response syndrome (SIRS), studies have found that γδT cells can prevent lung tissue damage by recognizing and eliminating inflammatory PMN (63). It is proved that γδT cells have cytotoxicity to activated macrophages and play an important role in the down-regulation of inflammatory response (64), which indicates that γδT cells are involved in the recovery of infection and can accelerate the recovery of infection. Another study found that γδT cells produce IFN-γ and anti-inflammatory cytokines (such as IL-10), which can inhibit the production of pro-inflammatory mediators (including IL-1, TNF-α and IL-8) in inflammatory cells in affected tissues. In addition to the ability to eliminate inflammatory cells, γδT cells can also play an additional role in protecting the integrity of host tissues and organs.

In the process of liver fibrosis, studies have shown that liver γδT cells, especially γδT1 subsets, play a significant protective role in the development of liver fibrosis. γδT cells can induce the apoptosis of HSC and inactivate activated HSC, so as to delay the process of liver fibrosis. In addition, γδT cells may inhibit the infiltration of inflammatory cells into liver tissue, and the lack of γδT cells will aggravate liver fibrosis, increase serum ALT levels, and accumulate intrahepatic white blood cells (65).

In terms of tumors, γδT cells may promote tumor development. Studies have found that CD39 γδTregs not only have a direct immunosuppressive function on effector T cells, but also secrete a large amount of IL-17A, TNF-α and GM-CSF.These cells may mobilize and recruit PMN-MDSCs into TME, thereby establishing an immunosuppressive network in colorectal cancer. In addition, under the induction of TGF-β1 secreted by tumor cells, cells produce more adenosine, which shows obvious immunosuppressive effect on CD4 ^+ ^T cells through adenosine-mediated pathway, and promotes tumor progression and metastasis (66–68). Another study found that Th17 γδT cells that produce IL-17 increase the expression of angiogenic factors VEGF-2 and ANG-2 in tumor sites, indicating that they promote tumor development in gallbladder cancer, ovarian cancer and breast cancer by enhancing angiogenesis (69).

It can be seen that γδT cells have a two-way immunomodulatory effect, which can not only mediate the immune response, but also produce immunosuppressive effects under certain conditions.

## 5. The role of γδT cell-mediated immune response in MIRI

There is a close relationship between γδT cells and IL-17. Through a variety of disease mouse model experiments, γδT cells, NK cells, neutrophils and other innate immune cells can produce IL-17.Early studies have found that γδT cells can also secrete IL-17 after receiving PMA/Ionomycin stimulation *in vitro* (70). As the most widely studied pro-inflammatory mediator in the IL-17 family, IL-17 A is involved in the occurrence and development of many infectious diseases, tumors and autoimmune diseases (71). γδT cells are the main source of IL-17A in the early immune response. The activation of γδT cells does not depend on the effect of antigen on TCR.IL-1 and IL-23 produced by activated dendritic cells can induce γδT cells to produce IL-17 (72). In the early stage of MIRI, IL-17A produced by γδT cells in the myocardium is the most important inflammatory cytokine. Apoptosis is considered to be an important mechanism of massive cell death in myocardial ischemia-re-perfusion (73). More and more evidence shows that the elements of innate immunity and adaptive immunity are involved in I/R injury (74). γδT cells play an important role in the immune response to myocardial ischemia-re-perfusion injury. γδT cells are T cells that perform innate immune functions. This heterogeneous cell includes multiple cell subsets and has multiple effector functions of producing cytokines and cytotoxicity (75–77). It has been found that the enhancement of immune response mediated by γδT cells aggravates the degree of myocardial ischemia-re-perfusion injury. In the early stage of ischemia-re-perfusion injury, the infiltration of a large number of neutrophils and monocytes/macrophages leads to a strong inflammatory response. At the same time, a large number of neutrophils and monocytes/macrophages produce and secrete various cytokines, chemokines and adhesion molecules, which ultimately aggravate tissue damage (78, 79).

### 5.1. γδt cells rapidly produce Il-17A

Due to the special pattern of antigen recognition and activation, γδT cells can immediately respond to various pathogens or (IL-1/IL-23), and produce a large amount of IL-17A within a few hours (80). IL-17A produces an immune response by gene-induced recruitment and migration of neutrophils. The IL-17 family is an important cytokine in the human body. In particular, IL-17A has been extensively studied and is mostly secreted by CD4^ + ^T cells. However, the inflammatory cytokine IL-17A during MIRI is mainly produced by γδT cells in the myocardium (81). Studies have shown that IL-17A in the myocardium is almost instantaneously increased after I/R (82), and the process of cytokine expression by γδT cells is a transient process. Although 70% of IL-17A is expressed by Th cells, the differentiation of naive CD4^ + ^T cells into Th17 cells takes a long time and cannot produce a large amount of IL-17A in a process similar to myocardial ischemia. Because the process of IL-17A production by γδT cells does not require pre-induction, γδT cells can quickly produce IL-17A and produce subsequent immune killing of cardiomyocytes. Thus, γδT lymphocytes are the main source of IL-17A.

### 5.2. IL-17A is the core cytokine of γδT-mediated immune response

The IL-17 cytokine family includes IL-17A, IL-17B, IL-17C, IL-17D, IL-17E and IL-17F. Among them, IL-17A was first discovered in clinical practice (83), and it is also the most widely studied cytokine in the family, and it is one of the most important pro-inflammatory cytokines (84, 85). The genes of IL-17A and IL-17F are located in the same chromosome region and are bound by the same complex IL17RA-IL17RC, so they have the highest structural homology and similar biological functions in the IL-17 cytokine family (86, 87). IL-17A, as a cytokine derived from activated T cells, is now considered to be a key pro-inflammatory cytokine in immune-mediated inflammatory diseases (88).Its mechanism is to recruit neutrophils and monocytes by producing chemokines to cause inflammation. IL-17A also plays an important role in promoting chronic inflammation and autoimmunity in mouse models (89–91). IL-17A and IL-17A-producing cells have become important targets for drug research to treat various forms of autoimmune and inflammatory diseases. Studies have shown that during MIRI, IL-17, as a special pro-inflammatory cytokine, participates in the occurrence of myocardial ischemia-re-perfusion injury by promoting cardiomyocyte apoptosis, recruiting neutrophil infiltration, and leading to myocardial remodeling, and is closely related to the pathogenesis of various cardiovascular diseases (92–94).

### 5.3. The role of Il-17A produced by γδT cells in MIRI

Innate immunity and adaptive immunity play an important role in the pathological process of MIRI. IL-17A acts as a bridge between innate immunity and adaptive immunity. IL-17A induces a typical inflammatory response through a strong gene-induced innate immunity, presenting a unique positional process in the immune response (95, 96). The recruitment and migration of neutrophils by IL-17A is a key process of myocardial I/R injury. IL-17RA and IL-17RC are key mediators of neutrophil recruitment and migration, which induce neutrophil production and production of neutrophil chemokines, including lipopolysaccharide-induced CXC chemokine(LIX) (97), cytokine-induced neutrophil chemoattractant (KC) and macrophage inflammatory factor protein-2 (MIP-2) -mediated neutrophil migration. IL-17A promotes EC to enhance the expression of neutrophil infiltration E-selectin and ICAM-1 (98), thereby promoting inflammatory response and aggravating myocardial ischemia-re-perfusion injury. In vitro studies have further confirmed that IL-17A has a direct pro-apoptotic effect on cardiomyocytes. When cardiomyocytes are exposed to hypoxia and oxidative stress, the apoptotic signaling pathway is activated, Fas mRNA and Bcl-2 family proteins are up-regulated, and the redox state changes, thereby regulating the Bax/Bcl-2 ratio to induce cardiomyocyte apoptosis. At the same time, caspase-3 apoptotic signaling pathway can also be regulated by IL-17A, thereby inducing cardiomyocyte apoptosis (99, 100).

**Figure 4:**
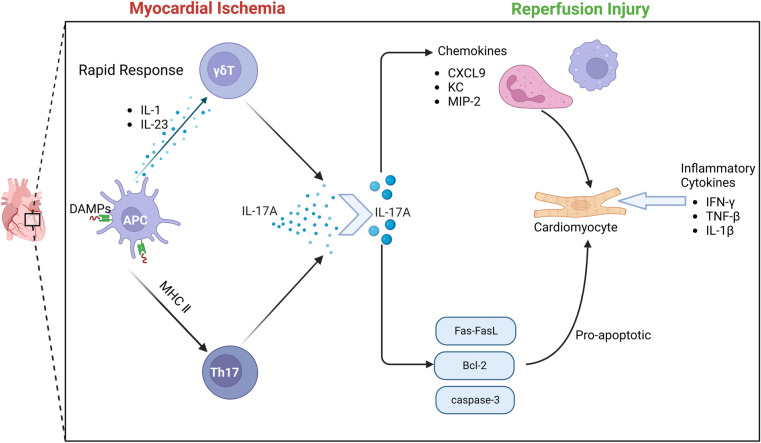
The role of γδT cell-mediated immune response in MIRI.

At the same time, studies have also confirmed that anti-IL-17A monoclonal neutralizing antibody treatment or IL-17A knockout significantly reduced neutrophil infiltration and inhibited cardiomyocyte apoptosis, significantly improving myocardial ischemia-re-perfusion injury. The supplement of exogenous IL-17A aggravated myocardial ischemia-re-perfusion injury. Another study found that IL-17A knockout or γδT cell ablation can improve the survival rate of mice after 7 days, indicating that IL-17A is involved in early myocardial ischemia-re-perfusion injury (101). In summary, the results show that the inflammatory cytokine IL-17 produced by γδT cells causes myocardial pathological damage by inducing cardiomyocyte apoptosis and neutrophil infiltration during myocardial ischemia-re-perfusion injury. Controlling the production of IL-17 may help reduce myocardial injury caused by I/R (102) Figure 4.

## 6. Therapeutic strategies based on γδT cell-mediated immune response in MIRI

Nowadays, the exact mechanism of MIRI has not been fully revealed, but more and more recent studies have confirmed that immune response plays a central role in various mechanisms of MIRI pathological process. Immune response affects the whole process of MIRI by activating innate immune system and adaptive immune system, as well as related immune cells. Among them, γδT cells play an important role in the MIRI immune response. Therefore, based on the immune response mechanism mediated by γδT cells, it can provide a new strategy for further treatment of MIRI.

Studies have shown that the adhesion and aggregation of neutrophils in myocardial tissue may be an important factor in mediating MIRI, and the induction of cytokines may play an important role. Under the stimulation of cytokine TNF, cardiomyocytes express higher levels of ICAM-1, which promotes neutrophil infiltration. Treatment with specific anti-ICAM-1 antibody can effectively protect myocardium and coronary vessels (103). Another study found that NKG2D can stimulate CD8 ^+^ T cells, γδT cells and NK cells to secrete cytokines (104), and NKG2D blockade can effectively reduce the expression of TnT, MPO, TNF and ICAM. NKG2D can also recognize a variety of ligands to play a variety of functions. NKG2D and its ligands effectively link innate immunity and adaptive immunity (105). Experiments have shown that NKG2D inhibitors can reduce the number of γδT cells that produce IL-17 after myocardial ischemia, and also inhibit the expression of IL-17 (106). NKG2D inhibitors can reduce the production of pro-inflammatory cytokines in myocardial tissue and effectively protect myocardial cells. Therefore, NKG2D inhibitors can be used as an effective means to alleviate MIRI.

In infectious or autoimmune diseases, γδT cells are the early main source of IL-17 production, which is regulated by RORγt expression. Since RORγt is the main transcription factor of IL-17 and is specific to IL-17, RORγt can be used as a therapeutic target for a variety of autoimmune diseases. It was found that RORγt inhibitors had a significant inhibitory effect on γδT cells and their secreted IL-17 in patients with spondyloarthritis and acute pancreatitis, and greatly improved the symptoms of patients. SR1001 is a traditional RORγt inhibitor. Its mechanism is to inhibit the activation and over-expression of γδT cells and inhibit the secretion of IL-17 by inhibiting RORγt transcription (107, 108). Therefore, the development of more types of RORγt inhibitors in the future can expand the therapeutic application in autoimmune diseases and provide new thinking for the prevention and treatment of MIRI.

An experiment found that inhibition of PI3Kδ can inhibit the production of IL-17 by some congenital and adaptive lymphocytes, such as γδT cells and MAITs to produce pro-inflammatory cytokines, thereby inhibiting downstream inflammation and tissue remodeling. Therefore, targeting PI3Kδ may become a new therapeutic approach for the treatment of MIRI mediated by γδT cells (109).

In addition to inhibitors, some extracts may also play an important role. Experimental studies have found that ATRA is an active metabolite of vitamin A, which has multiple effects on cell differentiation and survival by binding to two receptors RARs and RXRs (110, 111). ATRA can reduce antibody production in mice by inhibiting humoral immune responses *in vitro* and *in vivo*. In patients with Hashimoto's disease, ATRA attenuated the effect of γδT cells on the production of IgG, TPO-Ab and Tg-Ab by B cells. It can be seen that ATRA has a profound impact on the regulation of γδT cells. The regulation of ATRA can target activated γδT cells, which may promote the further activation and subsequent apoptosis of γδT cells through the mechanism of activation-induced cell death. Therefore, ATRA may be a potential regulator for the treatment of MIRI (112).

In recent years, traditional Chinese medicine is also playing an increasingly important role. It was found that hypericin, the extract of Hypericum perforatum, could inhibit the infiltration of γδT cells in spleen and lymph nodes. In another study, the reduction effect of hypericin on γδT cells was first elucidated, and it was found that hypericin reduced the expression and secretion of IL-17A in γδT cells. Hypericin inhibits the immune response of IL-17A-producing γδT cells and related cytokines by regulating the MAPK/Stat3 pathway (113). Based on the above drug mechanism, hypericin can provide a new treatment for clinical treatment of MIRI.

Psoriasis is a common chronic inflammatory disease. γδT cells accumulate in psoriatic lesions by rapidly secreting IL-17A, inducing and aggravating skin inflammation. It was found that the inhibitory effect of taxifolin on IL-17A may be related to the decrease of γδT cells. Taxifolin can significantly inhibit the activation of immune cells and down-regulate the level of IL-17 A gene in psoriatic skin lesions, and reduce the levels of IL-17 A, IFN-γ, IL-6 and other cytokines in peripheral blood. In addition, low-dose taxifolin also down-regulated the contents of chemokines MIP-1α and MCP-3, indicating that taxifolin can significantly inhibit lymphocyte migration and the effect of mononuclear macrophages on inflammatory lesions (114). Therefore, taxifolin can treat MIRI by inhibiting the activation of γδT cells and down-regulating the level of IL-17A.

Triptolide is a diterpene lactone compound extracted from Tripterygium wilfordii. Its pharmacological effects mainly include anti-tumor, anti-inflammatory and immune regulation. Experimental studies have found that triptolide can regulate the number of γδT cells and the expression level of cytokines. Triptolide can reduce the percentage of γδT cells in peripheral blood of arthritis model rats, and reduce the expression of γδT cells, TNF-α, IL-17 and IL-10 (115). Therefore, triptolide can be used for the treatment of MIRI by regulating the number of γδT cells and the expression level of IL-17A cytokines.

As the main active pharmacological component of ginseng, ginsenosides are often used to treat various diseases. GF2, as a ginseng extract, can play a variety of roles and functions in different tissues with low side effects, and has a variety of pharmacological effects in the treatment of inflammatory skin diseases, tumors, obesity and so on. GF2 has a significant anti-inflammatory effect. Experiments have shown that GF2 can reduce the infiltration of γδT cells, reduce the production of IL-17A, down-regulate the expression of CXCL1 in inflammatory skin tissues, and reduce neutrophil migration. In addition, GF2 also reduced ROS production in neutrophils. The anti-inflammatory effect of GF2 may be mediated by inhibiting the migration of γδT cells and the production of IL-17A and inhibiting the production of ROS and NET in neutrophils (116). Based on this possible potential mechanism, GF2 can be used as a suitable drug for the treatment of MIRI in the future.

**Figure 5:**
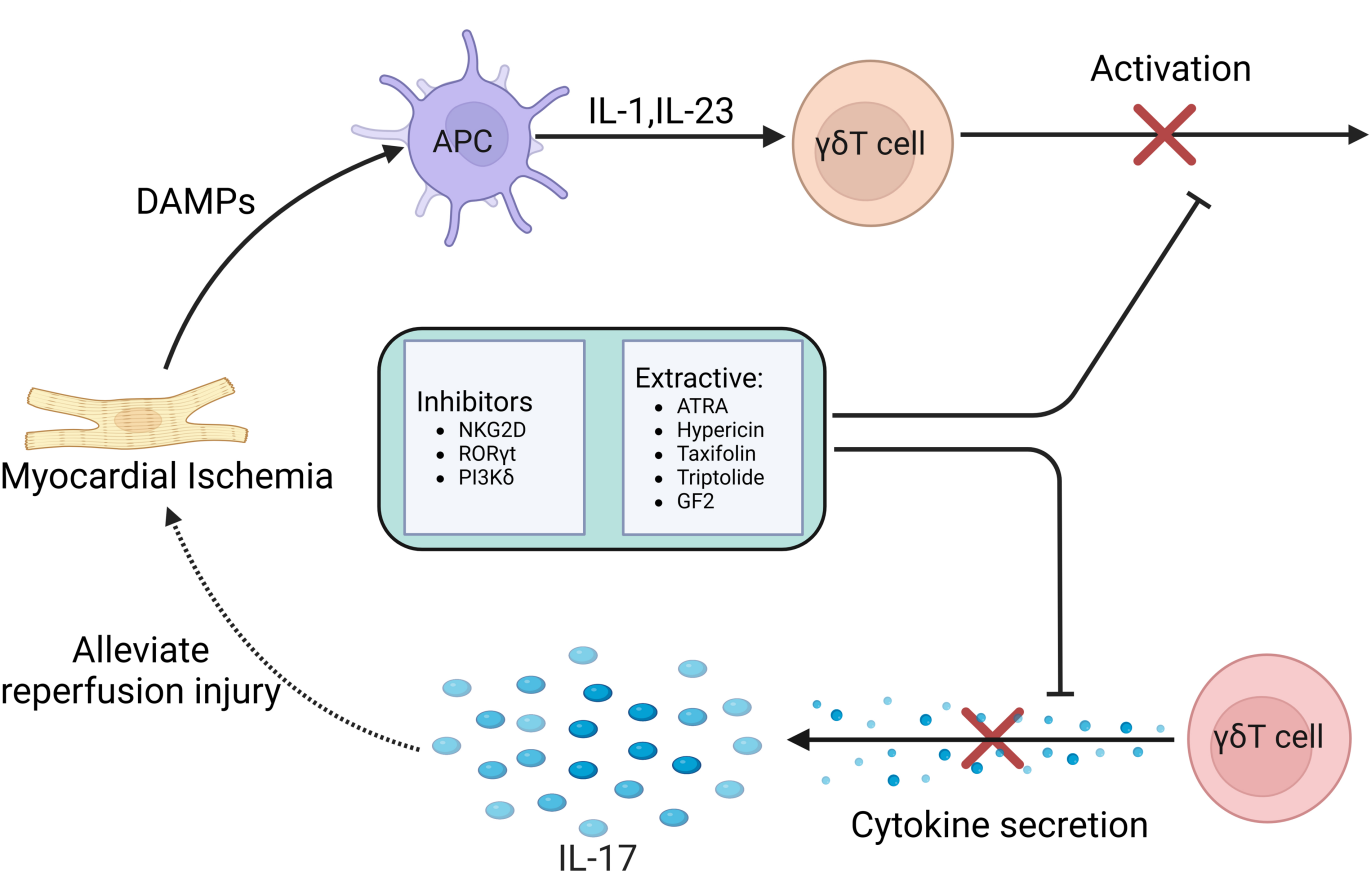
Therapeutic strategies based on γδT cell-mediated immune response in MIRI.

In summary, in the process of MIRI, γδT cells combine the characteristics of adaptive immunity with rapid innate immune response, so that they are in the initial stage of immune response. Current studies have confirmed that γδT cells-mediated immune response and the IL-17A produced by γδT cells play a key regulatory role in many infectious or autoimmune diseases. These treatments and drugs have significantly improved these diseases by inhibiting the activation of γδT cells and reducing the secretion of IL-17. Although there is still a lack of relevant clinical experimental studies on the role of MIRI, we believe that the immune response mediated by γδT cells can provide more therapeutic strategies for clinical prevention and treatment of MIRI in the future Figure 5.

## 7. Prospect of γδT cells in myocardial repair after ischemia-reperfusion injury

In the process of injury/repair of many diseases, the limited regenerative capacity of various tissues and organs has become a challenge in contemporary medicine. When the inflammation is controlled, the damaged tissue heals as the inflammation subsides, and the tissue structure and function recover. However, in some cases, fibrosis and scars are formed at the site of injury after inflammation subsides, which affects healing and leads to organ dysfunction. With continuous research, it has been proved that the immune system has a two-way regulatory role in the process of tissue and organ repair, which can lead to effective tissue regeneration, fibrosis and scar formation. Usually, myocardial injury is irreversible. When various cell types are delivered to the damaged heart, even if the delivered cells cannot survive, transplant or differentiate into functional muscle cells, the improvement of cardiac function is sometimes observed. The reason may be that the inflammatory response caused by activated macrophages temporarily enhances cardiac function.

The results of related studies on the innate immune system show that in the case of injury, activated fibroblasts, cardiomyocytes and various other immune cells release cytokines to polarize existing macrophages and chemokines, thereby recruiting more monocytes, activating and proliferating tissue-resident CCR2−macrophages (117, 118), which may protect and repair the damaged heart (119). Another study demonstrated that enhancing the activity of M2-like macrophages can promote cardiac function recovery after MIRI (120). Thus, CCR2 and CX3CR1 (M2-like) macrophages can be recruited by freezing and thawing/killing cells or local injection of zymosan (an effective stimulator of the innate immune system) to protect and repair damaged hearts.

The results of adaptive immune system showed that T cell infiltration may have pleiotropic effects on damaged myocardium (121). On the one hand, pro-inflammatory CD8 T cells are activated by dendritic cells to produce effective cytokines, including IL-17, IFN-γ and TNF-α, causing myocardial damage. In addition, CD8 T cells are essential for M1-like macrophage infiltration and secretion of pro-inflammatory cytokines and chemokines (122–124); and some CD4 T cells also show destructive effects in the injured heart. On the other hand, some infiltrating T cells have protective effects in damaged myocardium. A major role is to directly activate cardiac fibroblasts and induce fibrosis. The rapid pro-inflammatory response of CD4 T and CD8 T cells can enhance scar formation in the acute phase of injury, but may not be conducive to cardiac function recovery in the long run (125). In addition, dendritic cells can promote myocardial repair by coordinating regulatory T cells to polarize macrophages into M2-like phenotypes; in the experimental model of myocarditis, CD4 non-specific effector T cells have been shown to prevent post-inflammatory fibrosis. In addition, reducing the level of pro-inflammatory cytokines such as IL-17A can also inhibit the formation of fibrosis. CD4 Foxp3 T regulatory cells have been shown to be beneficial to wound healing, scar formation, inflammation regression and skeletal muscle injury repair after myocardial infarction.

Studies on the role of γδT cells in tissue repair have demonstrated that IL-17A produced by γδT cells plays an important role in promoting the proliferation of stem/progenitor cells (126). In muscle fiber injury/repair, it was found that IL-17A can directly promote the proliferation of MuSC, and the key to its repair mechanism is likely to produce IL-17A-mediated neutrophil accumulation through γδT cells to remove necrotic muscle fibers after muscle injury (127). In skin injury/repair, different doses of IL-17A play different roles in wound healing. Low or high doses of IL-17A are not conducive to the repair of skin wounds, while medium doses of IL-17A can effectively promote skin wound healing (128). The γδT cell subset in the skin immune system is usually called dendritic epidermal T cells (DETC). DETC is produced in the thymus during embryonic development and implanted into the epidermis to maintain a steady-state population. DETC has a characteristic dendritic morphology, which can monitor signs of injury or disease, and allow the proliferation and migration of DETC and keratinocytes when keratinocytes are damaged, which is essential for wound healing (129, 130).

γδT cells act as a bridge between innate immunity and adaptive immunity, and have a two-way immune effect. Targeted immunoregulatory γδT cells may be a potential treatment for myocardial ischemia-re-perfusion injury. It can promote the recovery after injury and prevent the secondary injury of myocardial cells after reperfusion. Inhibit fibroblast activation and reduce adverse remodeling. With the continuous deepening of research to further determine the targeted immunomodulator, it can have a specific effect on the components of the immune response, which may be an attractive direction for future clinical treatment of myocardial ischemia-re-perfusion injury.

## 8. Discussion

In summary, innate immune response and adaptive immune response as the body's defense system play an important role in cardiovascular disease. However, the regulation of the immune system is very complex in different physiological and pathological backgrounds. A large number of studies have shown that the regulation of the immune system in MIRI is a cardiac protection mechanism to protect it from different types of damage, but in some cases, excessive immune response will aggravate the damage to the body. Among them, γδT cells play an important role in MIRI. The activation and release of inflammatory factors is an important cause of myocardial ischemia-re-perfusion injury. Inhibiting the release of inflammatory factors can maintain the stability of cardiomyocytes. Although the proportion of γδT cells in the total T cell population is small, γδT cells have become an important regulator of early immune response and have become a key immune cell type in the prevention and treatment of cardiovascular diseases.As a bridge between innate immune response and adaptive immune response, γδT cells have the characteristics of non-MHC-restricted recognition of receptors, rapid activation, and bidirectional immunity. They have received more and more attention in cardiovascular immunotherapy. Targeted immunoregulatory γδT cells may be a potential treatment method, which is conducive to promoting the recovery after myocardial injury, preventing secondary damage to cardiomyocytes after reperfusion, inhibiting fibroblast activation, and reducing adverse remodeling. Therefore, it can be used as a new idea for the treatment of myocardial ischemia-re-perfusion injury. However, the current research on the treatment of myocardial ischemia-re-perfusion injury based on immune response and γδT cells is still limited, and more research is needed. For example, the combination of optimized immune regulation detection methods and disease animal models can eventually make γδT cells become targeted personalized immunotherapy, which will help us to diagnose and treat diseases more accurately and provide new ideas and methods for clinical prevention and treatment of cardiovascular diseases.

## Data Availability

The original contributions presented in the study are included in the article/Supplementary Material, further inquiries can be directed to the corresponding author.
